# Direct cell fate conversion of human somatic stem cells into cone and rod photoreceptor-like cells by inhibition of microRNA-203

**DOI:** 10.18632/oncotarget.9882

**Published:** 2016-06-07

**Authors:** Soon Won Choi, Ji-Hee Shin, Jae-Jun Kim, Tae-Hoon Shin, Yoojin Seo, Hyung-Sik Kim, Kyung-Sun Kang

**Affiliations:** ^1^ Adult Stem Cell Research Center, College of Veterinary Medicine, Seoul National University, Seoul 08826, Republic of Korea; ^2^ Research Institute for Veterinary Science, College of Veterinary Medicine, Seoul National University, Seoul 08826, Republic of Korea; ^3^ Pusan National University School of Medicine, Busan 49241, Republic of Korea; ^4^ Biomedical Research Institute, Pusan National University Hospital, Busan 49241, Republic of Korea

**Keywords:** miR-203, photoreceptor, neural retina, AESC, UCB-MSC

## Abstract

Stem cell-based photoreceptor differentiation strategies have been the recent focus of therapies for retinal degenerative diseases. Previous studies utilized embryonic stem (ES) cells and neural retina differentiation cocktails, including DKK1 and Noggin. Here, we show a novel microRNA-mediated strategy of retina differentiation from somatic stem cells, which are potential allogeneic cell sources. Human amniotic epithelial stem cells (AESCs) and umbilical cord blood-derived mesenchymal stem cells (UCB-MSCs) treated with a retina differentiation cocktail induced gene expressions of retina development-relevant genes. Furthermore, microRNA-203 (miR-203) is abundantly expressed in human AESCs and human UCB-MSCs. This miR-203 is predicted to target multiple retina development-relevant genes, particularly DKK1, CRX, RORβ, NEUROD1, NRL and THRB. The inhibition of miR-203 induced a retina differentiation of AESCs and UCB-MSCs. Moreover, successive treatments of anti-miR-203 led to the expression of both mature photoreceptor (PR) markers, rhodopsin and opsin. In addition, we determined that CRX, NRL and DKK1 are direct targets of miR-203 using a luciferase assay. Thus, the work presented here suggests that somatic stem cells can potentially differentiate into neural retina cell types when treated with anti-miR-203. They may prove to be a source of both PR subtypes for future allogeneic stem cell-based therapies of non-regenerative retina diseases.

## INTRODUCTION

Neural retina degeneration is a human eye disorder that leads to the loss of vision due to the decay of various cell types in the neural retina [1]. Cone-rod dystrophy is one type of neural retina degenerative disease that causes a depletion of the outer nuclear layer, which consists of cone and rod PR cells. Both types of PRs are involved in visual signal transduction, along with retinal pigmented epithelial (RPE) cells, as confirmed by their response to light. Once the PR or RPE cells are lost, they cannot be regenerated naturally. Therefore, the use of stem cells for vision restoration has recently been under intense investigation.

ES cells and induced pluripotent stem (iPS) cells are a useful source for the production of neural retina components for tissue regenerative strategies for retinal pathologies. In fact, many ES/iPS cell studies have focused on the production of retinal progenitor cells or mature cell types, such as PR cells or RPE cells. Under appropriate differentiation conditions, human ES and iPS cells can be differentiated into retinal progenitor cells or optic vesicle-like structures, which can subsequently differentiate into the various retinal subtypes [2, 3]. Other studies have reported that both cone and rod PR cells can be derived from human ES and iPS cells [4–7]; furthermore, mouse ES cell aggregates were shown to form the optic cup structure, which is composed of the neural retina and RPE [8]. Several key publications for retina differentiation used a cocktail of growth factors, such as IGF-1 and bFGF, and exogenous Wnt and BMP antagonists, such as Dkk1 and Noggin [4, 6, 8]. In particular, the results revealed that the effective differentiation of neural retina progenitor cells could be promoted by the addition of DKK1 and Noggin. In support of these results, both the Wnt and BMP signaling pathways are known to antagonistically influence neural development [9–11].

Apart from the use of these growth factors, neural differentiation or conversion is also possible with the aid of transcription factors, which lead lineage-specific development and ultimately determining the cell's fate. A break-through study by Vierbuchen *et al*. demonstrated that somatic cells, such as fibroblasts, could be converted into neurons or neural stem cells using a combination of transcription factors [12–14]. Cell type-specific transcription factors for neural retina development are well-defined, not only as markers for each major stage of retina development but also with respect to their roles in retina specification. Six transcription factors are known to play key roles in PR specification, specifically the PR precursor markers OTX2, CRX and RORβ, the cone PR marker THRB and the rod PR markers NR2E3 and NRL [15]. The PR marker OTX2 determines PR cell fate, whereas both CRX and RORβ induce the terminal differentiation of PR cells [16–19]. The cell fate specification between a cone or rod PR subtype is controlled by several factors; NRL and its target NR2E3 prompt a retinal progenitor cell toward a rod PR cell fate [20–22], while THRB leads to the development of an M cone PR cell from a dominant S cone [23].

To regulate the expression of cell type-specific transcription factors, retroviral or lentiviral infection systems are most commonly used because of their ease of use, practical application and induction of long-term stable gene expression. In previous studies using such viral-mediated transduction methods, it is reported that neurons, including dopaminergic and motor neurons, can be generated from fibroblasts via the regulation of transcription factor expression [24–26]. Although it is a powerful technique, there are limitations, such as the genomic integration of exogenous sequences that occurs randomly because of the viral-mediated transduction; this is a critical disadvantage for clinical applications. An alternative class of regulatory molecules is the microRNA (miRNA) family, which is well-characterized as a post-transcriptional regulator of gene expression. miRNAs target the 3′-UTRs of multiple mRNAs at the same time and fill a repressive role in the translation process [27, 28]. A recent report has demonstrated that miR-9* and miR-124, which are expressed in post-mitotic neurons, mediate the conversion of fibroblasts into neurons [29].

Somatic stem cells have some advantages over ES cells for cell-based regenerative therapies. For example, ES cell-derived neural progenitor cells could give rise to teratomas in a host because of their unlimited self-renewal ability, while somatic stem cells cannot because of their limited self-renewal ability [30]. However, it is important to note that ES cells can proliferate *in vitro* and *in vivo* following transplantation. In addition, somatic stem cells are derived from an adult and can provide patient-specific cell therapy without the risk of transplant rejection by the immune system.

**Figure 1:**
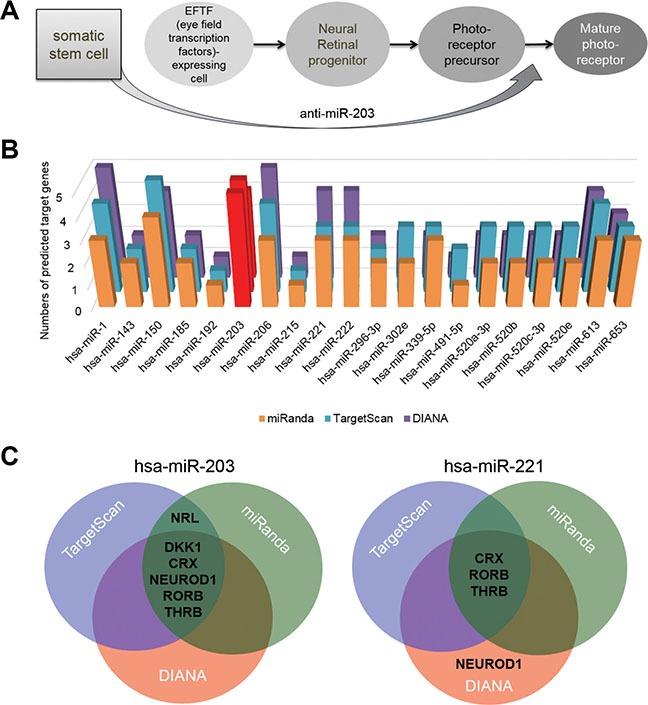
miR-203 targets retina development-relevant genes (**A**) A schematic of the neural retina differentiation of ES cells into mature PRs with markers that indicate intermediate developmental steps. The hypothesis of retina differentiation from somatic stem cells directly by treatments of anti-miR-203 was shown. (**B**) The number of predicted targets for the most prominent 20 miRNAs were measured by using the target prediction program *miRanda*. Targets were selected among seven representative retina development-relevant genes (**C**) Predicted target genes of both candidate miRNA, miR-150 and miR-203, were illustrated as a Venn diagram. The overlapping area of the Venn diagram represents putative targets predicted by three different miRNA target prediction programs.

As mentioned previously, several studies have induced ES cells to undergo neural retina differentiation [3, 4, 6]. In these studies, ES cells were differentiated into PRs through several key stages, such as the eye field transcription factor (EFTF)-expressing cell, neural retina progenitor and PR precursor (Figure 1A). In our previous studies, we demonstrated proof-of-principle a direct cell fate conversion of somatic stem cells into RPE using a miRNA-based strategy without any growth factors [31]. To apply this miRNA-based strategy to a generation of PRs, we attempted to directly differentiate somatic stem cells into PR cells using the treatment of a single miRNA inhibitor. In this study, we show that anti-miR-203 treatment can mediate the differentiation of somatic stem cells into neural retina cell types, particularly PR cells.

## RESULTS

### Photoreceptor differentiation of somatic stem cells by a cocktail of recombinant proteins

Prior to attempting the differentiation by using a single miRNA, we assessed whether somatic stem cells can directly convert into neural retina cell types. First of all, we cultured AESCs and UCB-MSCs with a neural retina differentiation cocktail including Dkk1, Noggin, IGF-1 and bFGF (Supplementary Figure S1A). After 28 days, the round-shaped AESCs subsequently began to exhibit a neuron-like morphology (Supplementary Figure S1B). By the end of the differentiation, neural retina cell markers, e. g. OPN1MW, NRL, RHO, CHX10, were expressed in UCB-MSCs, whereas not in AESCs (Supplementary Figure S1C). Of these markers, two key rod PR genes NRL and RHO showed a relative high expression level in the cocktail-induced PR-like cells than in UCB-MSCs (Supplementary Figure S1D–S1E). The retina differentiation induction of AESCs and UCB-MSCs showed cell source-dependent consequences, i.e. increased expressions of two rod PR markers NRL and Rhodopsin in UCB-MSCs but not in AESCs. Altogether, these data indicated that a cocktail of defined proteins could barely differentiate UCB-MSCs into neural retina cell types.

### miR-203 targets multiple retina development-relevant genes

We hypothesized that a single miRNA targets multiple retina development-relevant genes and is expressed at a lower level in retina than in somatic stem cells. To investigate this hypothesis, we assessed the retina-relevant miRNAs by using miRNA target prediction programs: *TargetScan*, *DIANA* and *miRanda*. From this assessment, we first selected the most prominent 20 miRNAs, which are predicted to target a key retina development-relevant gene CRX. We next predicted the targets of each of the 20 miRNAs using miRNA target prediction programs; we specifically looked at seven retina development-relevant transcription factors, such as CRX, OTX2, NEUROD1, RORβ, NRL, THRB and NR2E3, and a retina induction factor DKK1 (Figure 1B and Supplementary Table S1). Of the 20 identified miRNAs, miR-203 and miR-221 are two most highly expressed miRNAs. As shown in the Venn diagram, three different miRNA target prediction programs predicted that the miR-203 precisely targets the six genes without OTX2 and NR2E3 genes (Supplementary Tables S2–S4), whereas miR-221 targets only RORB and THRB genes by three target prediction programs (Figure 1C). The results of target predictions significantly indicated miR-203 as a retina-relevant miRNA. Thus, we assumed that the inhibition of miR-203 could induce the neural retina differentiation of somatic stem cells.

### Transfections of a miR-203 inhibitor can induce expressions of retina development-relevant genes in AESCs

**Figure 2:**
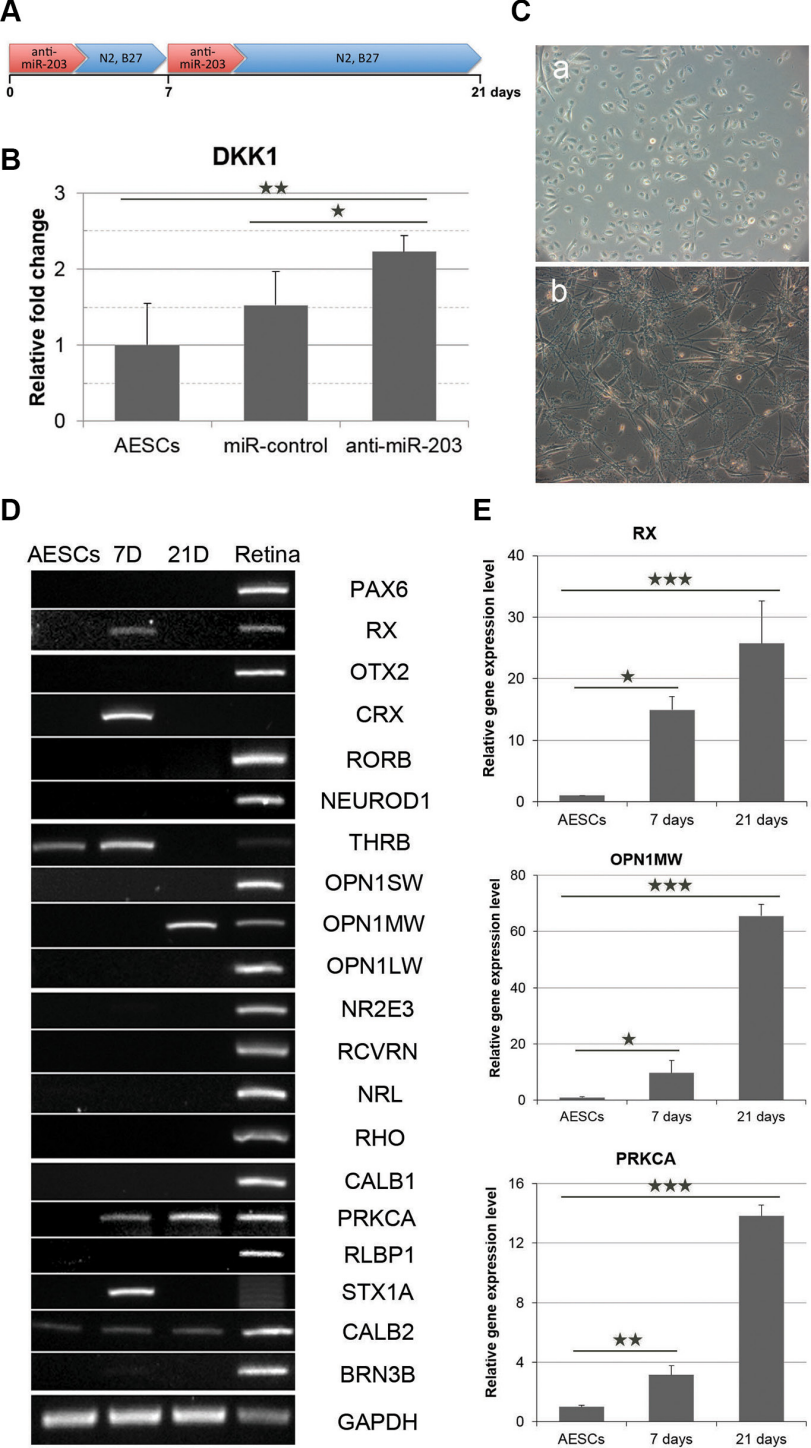
miR-203 inhibition induces the initial differentiation of AESCs into neural retina (**A**) A schematic of anti-miR-203 treatment during the retina differentiation of AESCs for 21 days. (**B**) A quantitative RT-PCR analysis shows the relative gene expression level of DKK1 at 3 days after the transfection of anti-miR-203. (**C**) Phase-contrast images of AESCs during anti-miR-203-mediated differentiation as shown at the day 0 (a) and days 21 (b). (**D**) A RT-PCR analysis shows the change in the retina-specific gene expression pattern after 7 days (7D) and 21 days (21D) of differentiation compared to normal human retina tissue (Retina). (**E**) The gene expression level of RX, OPN1MW and PRKCA during the initial differentiation as assessed by a quantitative RT-PCR analysis. *P < 0.05; **P < 0.01.

According to previous target predictions, we treated somatic stem cells with anti-miR-203 to block the function of mature miR-203 (Figure 2A). Three days after a transfection of anti-miR-203, the expression level of DKK1 was increased by two-fold (Figure 2B). Based on these data, we cultured AESCs for 21 days following transfections of anti-miR-203. Like the cocktail-induced retina differentiation, AESCs exhibited a morphological change toward a neuron-like shape (Figure 2C). During miRNA-induced retina differentiation, the gene expression profile of AESCs was gradually changed. A neural retina progenitor marker RX, a PR precursor marker CRX and an early cone PR marker THRB were expressed in AESCs 7 days after the first transfection (Figure 2D). After 3 weeks, the mature cone PR marker OPN1MW was subsequently expressed. In addition, gene expression levels were quantitatively assessed in these cells, and we observed that the expression levels of RX, OPN1MW and PRKCA were increased 25.7, 65.4 and 13.8 fold, respectively (Figure 2E). These data indicated that a miR-203 inhibitor could induced expressions of several retina development-relevant genes including miR-203 targets, DKK1, CRX and NRL, following transfections of anti-miR-203.

### A miRNA inhibitor induces retina differentiation of somatic stem cells

**Figure 3:**
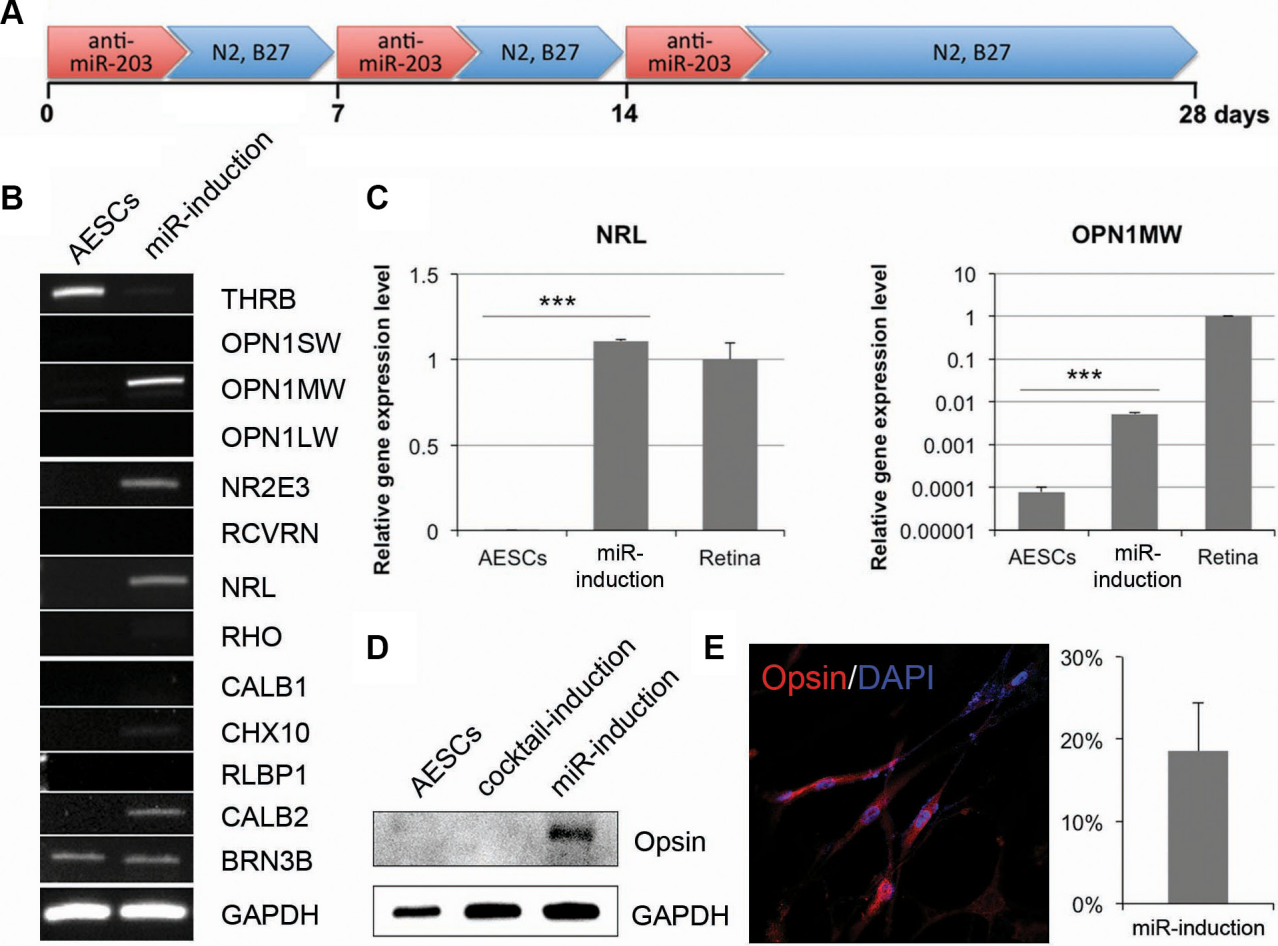
The long-term treatment of anti-miR-203 can induce retina maturation of AESCs (**A**) A differentiation scheme of successive treatments of anti-miR-203 shows the neural retina differentiation of AESCs over the course of 28 days. (**B**) A RT-PCR analysis shows the retina-specific gene expression pattern in AESCs and the miRNA-induced PR-like cells. (**C**) A quantitative RT-PCR analysis shows the relative gene expression level of OPN1MW and NRL compared to human retina tissue (Retina). (**D**) A western blot analysis shows the expression of Opsin in the PR-like cells, differentiated by cocktail or anti-miR-203. (**E**) The miR-induced PR-like cells expressed Opsin as shown by immunocytochemistry. The frequencies of expression are shown. Cell nuclei are stained with DAPI.

As above mentioned, a single transfection of anti-miR-203 was not enough to induce the expression of mature PR markers (Figure 2D). We next attempted to treat anti-miR-203 for the retina maturation. To address this, we cultured AESCs for 28 days following three successive transfections of anti-miR-203 (Figure 3A). At the end of the differentiation process, several genes were up-regulated in AESCs, namely the PR markers OPN1MW, NR2E3 and NRL (Figure 3B). Using quantitative RT-PCR analysis, the gene expression pattern of two PR markers NRL and OPN1MW in AESCs was investigated in detail (Figure 3C). Both PR markers were highly expressed to a significant extent in the miR-induced PR-like cells. In particular, the expression level of NRL was as high as the level found in human retina tissue. Moreover, a key cone PR marker Opsin was expressed in the miR-induced PR-like cells (Figure 3D–3E). Interestingly, the opsin was expressed to a greater extent in anti-miR-203-differentiated cells than in the cocktail-induced PR-like cells (Figure 3D). After the retina maturation of AESCs, the miR-induced PR-like cells exhibited a morphology similar to neurons and an increased expression rate of 18.5% (Figure 3E). Thus, successive treatments of a miR-203 inhibitor can induce the retina maturation of AESCs into the cone PR subtype.

**Figure 4:**
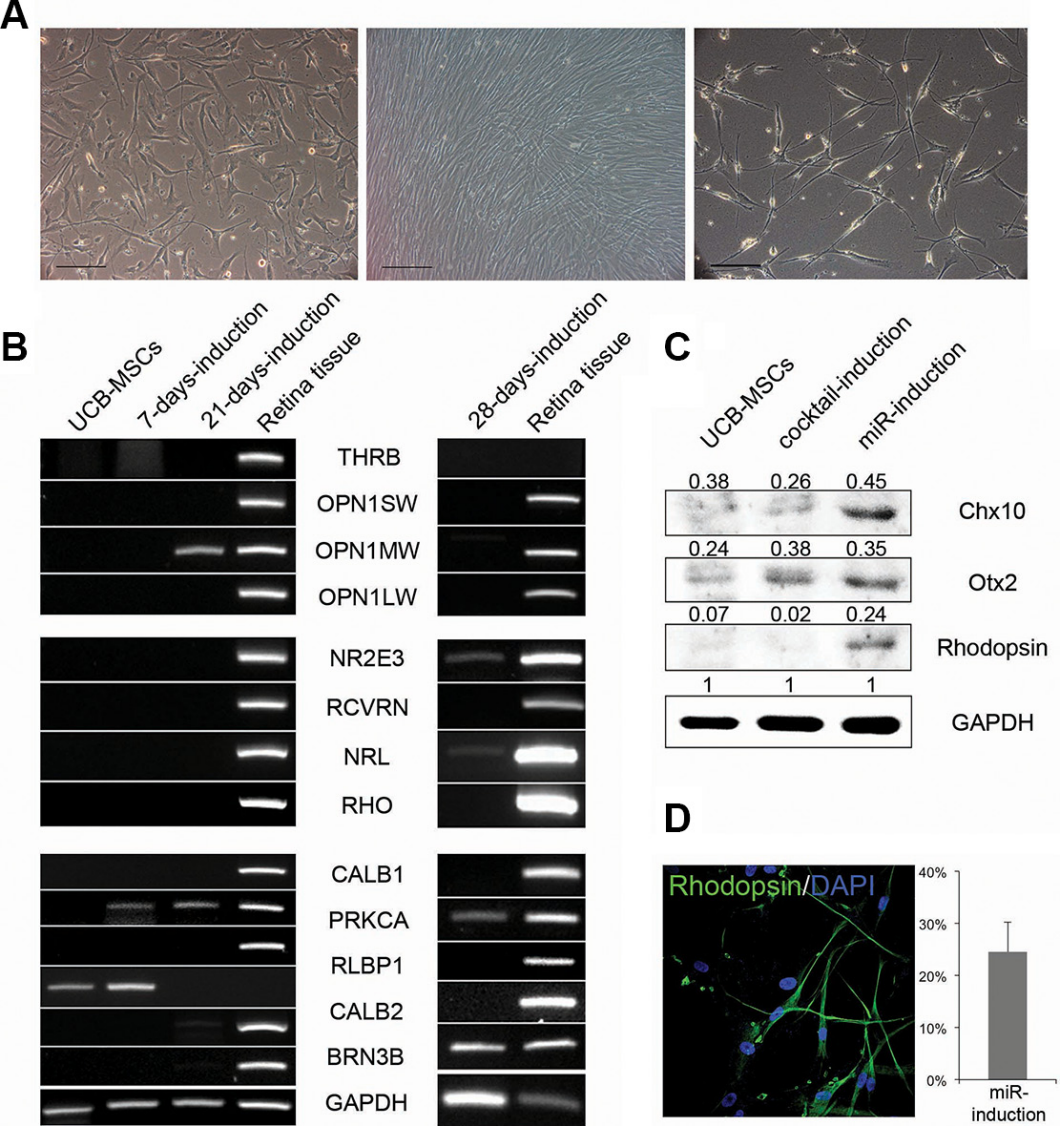
UCB-MSCs can also give rise to neural retina subtypes (**A**) Phase-contrast images of UCB-MSCs during the retina differentiation are shown at the day 0 (left), 7 (middle) and 28 (right). (**B**) A RT-PCR analysis shows the retina-specific gene expression pattern during the initial differentiation and the retina maturation compared to human retina tissue. (**C**) A western blot analysis indicates the expression of Chx10, Otx2 and Rhodopsin in UCB-MSCs and the cocktail-induced and miRNA-induced PR-like cells. (**D**) The miRNA-induced PR-like cells expressed Rhodopsin as shown by immunocytochemistry. The frequencies of expression are shown. Cell nuclei are stained with DAPI.

Then, we further analyzed the miR-induced retina maturation in UCB-MSCs. 21 days after treatments of anti-miR-203, morphologies of the cells were turned to a neuron-like shape (Figure 4A). The gene expression levels of retina maturation-relevant genes were gradually changed following transfections of anti-miR-203 (Figure 4B). Specifically, a cone PR marker OPN1MW and both rod PR markers NR2E3 and NRL were up-regulated in a time dependent manner. Another rod PR marker, rhodopsin, was detected in the miR-induced PR-like cells using western blot analysis and immunocytochemistry (Figure 4C–4D). The UCB-MSC-derived PR-like cells expressed rhodopsin at a rate of 24.5%. Although the retina differentiation of both somatic stem cell types was induced by treatment of a single miRNA inhibitor, they exhibited a retina maturation in a cell source-dependent manner.

### miR-203 directly regulates retina development-relevant genes

**Figure 5:**
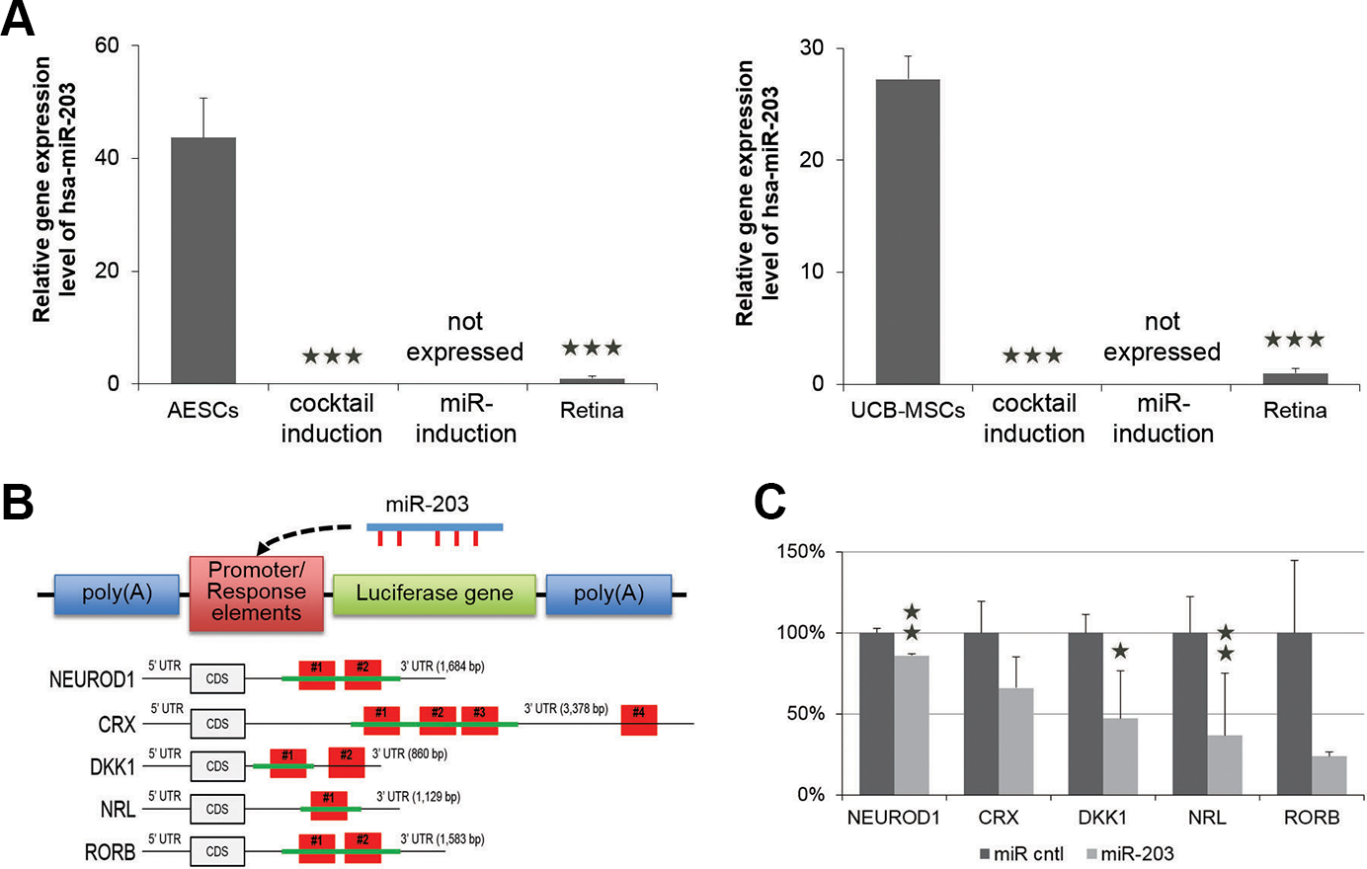
miR-203 directly targets genes involved in neural retina development (**A**) A quantitative RT-PCR shows the relative expression level of miR-203 in somatic stem cells that were undifferentiated, differentiated by a neural retina-specific cocktail of exogenous factors (DKK1) or differentiated by anti-miR-203 compared to human retina tissue (Retina). (**B**) Schematics of the reporter constructs of *pmirGLO Dual-Luciferase* vector. Response elements consist of the cloned sequence (green lines) from 3′UTR of predicted target genes including one or multiple binding sites (red boxes). This reporter construct could be targeted by miR-203. (**C**) The relative luciferase activity in *293T* cells expressed as the fold change of Firefly to Renilla after miR-203 treatment. These ratios were normalized to the value of each reporter construct. *P < 0.05; **P < 0.01.

To better understand the effects of anti-miR-203 in an induction of retina differentiation, we investigated the relative expression level of miR-203 in the somatic stem cells, the cocktail-induced PR-like cells, the miR-induced PR-like cells and retina tissues. Compared to the retina tissue, the expression level of miR-203 in AESCs and UCB-MSCs was significantly higher, specifically 44-fold and 27-fold, respectively (Figure 5A). Interestingly, the expression levels of miR-203 in both somatic stem cell types following retina differentiation were significantly decreased compared to the retina tissue. Moreover, the expression of miR-203 after miR-induced retina differentiation was not detectable. It appears that the retina differentiation using cocktail and anti-miR-203 diminished miR-203′s presence in somatic stem cells and subsequently induced the retina differentiation.

As described above, anti-miR-203 up-regulated retina development-relevant genes DKK1, CRX and NRL, which are predicted as targets of miR-203. To provide evidence for direct targets of miR-203, we performed a luciferase assay and analyzed, whether treatments of miR-203 can directly reduce the expression of three target genes DKK1, CRX and NRL. First, we separately cloned the 3′-untranslated regions (3′-UTRs) from NEUROD1, CRX, DKK1, NRL and RORB mRNAs into the *pmirGLO Dual-Luciferase* vector (Figure 5B and Supplementary Tables S2–S4). The 3′-UTR sequence of the NEUROD1 transcript contains two predicted miR-203 binding sites (#1 at nucleotides 511-532 and #2 at nucleotides 683–704). The 3′UTR sequence of the CRX transcript contains four predicted miR-203 binding sites (#1 at nucleotides 317–345, #2 at nucleotides 360–388, #3 at nucleotides 638–666 and #4 at nucleotides 3158–3179). The 3′-UTR sequence of the DKK1 transcript contains two predicted miR-203 binding sites (#1 at nucleotides 346-368 and #2 at nucleotides 549-570). The 3′-UTR sequence of the NRL transcript contains one predicted miR-203 binding site (#1 at nucleotides 446-467). The 3′-UTR sequence of the RORB transcript contains two predicted miR-203 binding sites (#1 at nucleotides 944-965 and #2 at nucleotides 1058-1079). We cloned constructs with all binding sites into the luciferase vector for NEUROD1, NRL and RORB, and we cloned constructs with part of binding sites into the luciferase vector for CRX and DKK1.

Next, we co-transfected this luciferase vector including binding sites and a reporter construct together with miR-203 into HEK-293FT cells to determine whether the predicted binding sites in each 3′-UTR were responsible for gene expression. The relative luciferase activities of the predicted target genes NEUROD1, CRX, DKK1, NRL and RORB were decreased to 85.8 ± 1.4, 65.8 ± 19.2, 47.0 ± 8.6, 36.6 ± 6.6 and 23.8 ± 24.2%, respectively. Of three genes, NRL, DKK1 and NEUROD1 showed that the luciferase activity was significantly suppressed in the presence of miR-203 (Figure 5C). These data demonstrate that miR-203 directly interacts with the predicted target binding sites of miR-203 in the 3′-UTRs of target genes DKK1, CRX, NRL, NEUROD1 and RORB. Furthermore, the down-regulation of miR-203 targets explains the reason for the induction of the retina differentiation.

## DISCUSSION

Recent strategies for neural retina differentiation utilize recombinant proteins or a viral system to express multiple neural retina developmental factors. In this project, we show that miR-203 is expressed abundantly in somatic stem cells and that anti-miR-203 can induce the expression of neural retina developmental factors. With successive treatments of anti-miR-203, somatic stem cells can be differentiated into PR cell types. These findings facilitate non-viral strategies for cell differentiation for basic research; moreover, this strategy may have therapeutic applications in non-regenerative retina diseases.

Several studies have reported the retinal development-specific expression and function of miRNAs. Conte *et al*. have demonstrated that miR-204 is expressed in the lens placode and presumptive RPE during early medaka development [32]. They described that miR-204 directly targets Meis2 and regulates the Meis2/Pax6 pathway in both lens and retina development. Similarly, other miRNAs have been shown to exhibit retina-specific expression and function. miR-24a is expressed in the developing neural retina of *Xenopus tropicalis* and contributes to the eye size through the regulation of apoptosis in the retina [33]. In addition, miR-124a was found to be highly expressed during eye development from the late stages of the embryo to adulthood; miR-124a was also shown to be a post-transcriptional regulator that controls eye morphogenesis and neurogenesis [34]. These retina development-specific miRNAs caused global consequences in the eye development of vertebrates by targeting early eye developmental genes, such as Meis2, Lhx2 and Otx2.

**Figure 6:**
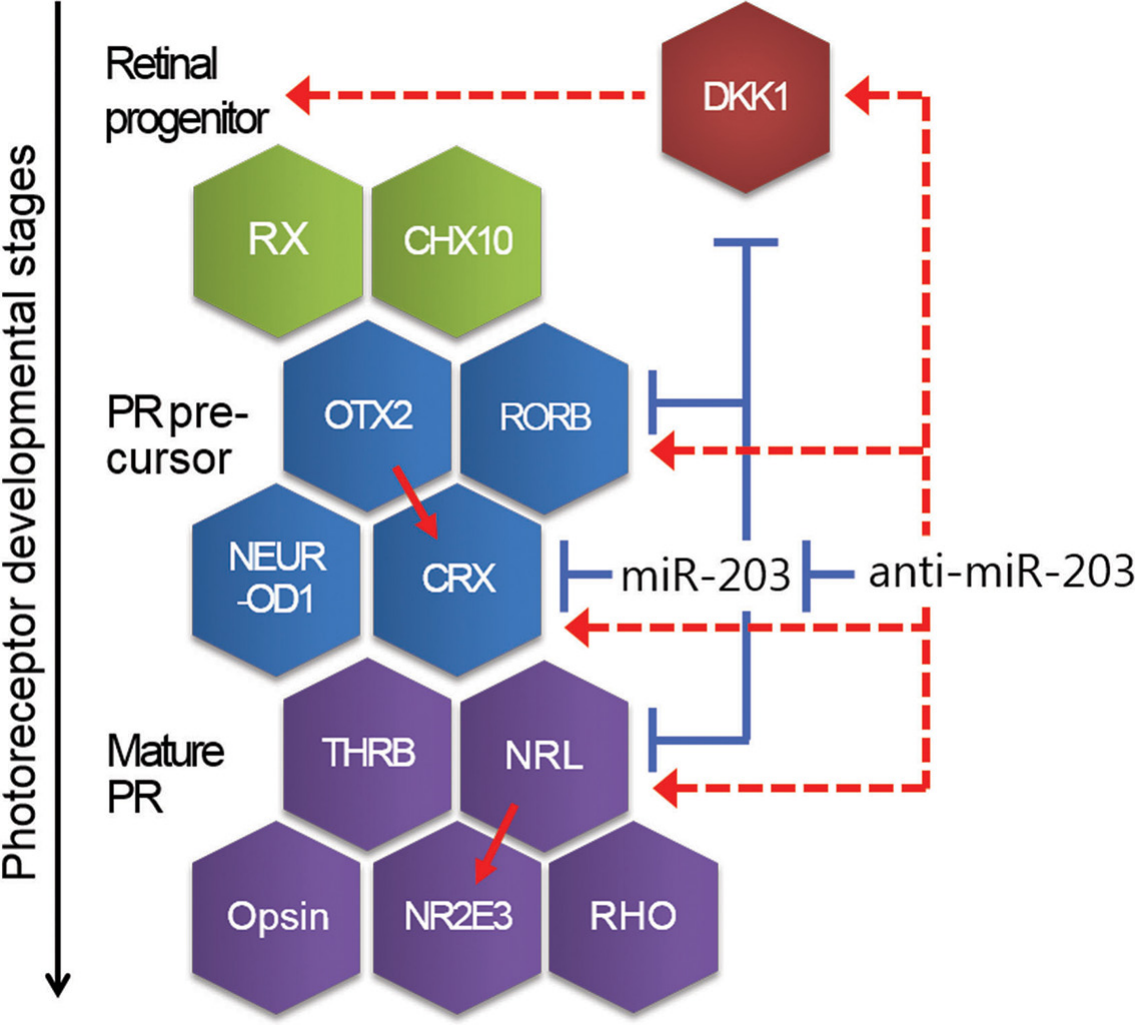
A schematic showing the developmental steps and genes relevant for neural retina development Mature miR-203 is involved in neural retina differentiation as a repressor of genes, such as DKK1, CRX and NRL. Conversely, Anti-miR-203 up-regulates those target genes through inhibiting of miR-203. Red line, promoting. Blue line, inhibiting. Dotted line, indirect regulating.

Unlike the miRNAs discussed above, a novel finding of miR-203′s role in neural retina differentiation suggests that miR-203 targets six retina development-specific factors at various stages, such as the retina progenitor, PR precursor and mature PR (Figure 6). Of these factors, a transcription factor, CRX, and an orphan nuclear receptor, RORβ, are important for the PR specification of PR precursors. CRX-deficient mice and RORβ-deficient mice lack PR outer segments; furthermore, both factors are known to contribute synergistically to the activation of S opsin in cone PR development [18, 19, 35]. For PR cell fate determination, a basic motif-leucine zipper transcription factor, NRL, and a ligand-regulated transcription factor, THRB, play crucial roles. S cone PR cells are enriched in NRL-deficient mice and THRB-deficient mice [23, 36]. NRL directs the conversion of dominant S cone PR cells to a rod cell fate, whereas THRB induces dominant S cone PR cells to adopt an M cone cell fate. Thus, controlling the expression of miR-203 with an anti-miR-203 affects the expression of these crucial neural retina development genes and guides the differentiation of somatic stem cells toward a neural retina cell fate.

As in the results mentioned, the retina maturation of both AESC- and UCB-MSC-derived cells was induced in a cell source-dependent manner. In the near future, we are planning to investigate the mechanistic study of miR-203 -mediated direct cell fate conversion in somatic cells. It is interesting and would provide additional information about cell source-specific maturation. In conclusion, we have shown that miR-203 is abundantly expressed in somatic stem cells and represses three retina development-specific factors. Upon treatment with anti-miR-203, retina development genes were expressed in these cells, and the cells were consequently converted to PR cell types. Finally, our study shows that a miRNA inhibitor can be a useful tool to direct cell differentiation by regulating multiple genes, which are suppressed by key miRNAs.

## MATERIALS AND METHODS

### Cell cultures

The human AESCs and human UCB-MSCs were primary cultured as previously described [37, 38]. AESCs show a positive expression pattern with CD4, −24, -29, -49b, -73, -90 and -105, as well as a negative expression pattern with CD14, -31, -34 and -45. These cells were maintained in *Keratinocyte - Serum Free Medium*(SFM, Invitrogen) with *Supplements For Keratinocyte - SFM* (Invitrogen), 10% fetal bovine serum (FBS, Invitrogen), 100 U/ml penicillin (Invitrogen) and 100 U/ml streptomycin (Invitrogen). UCB-MSCs show a positive expression pattern with CD24, -29, -44, -73, -90 and -105, as well as a negative expression pattern with CD10, -14, -31, -33, -34, -45, -62p and -133. These cells were cultured in *Endothelial Cell Basal Medium-2* (EBM-2, Lonza) with *EGM-2 SingleQuots* (Lonza) 10% FBS, 100 U/ml penicillin and 100 U/ml streptomycin. For the induction of neural retina differentiation, we used passage numbers from 2 to 5 for AESCs and from 8 to 15 for UCB-MSCs.

### Induction of neural retina differentiation

For the induction, the media were changed to the differentiation medium containing N-2 (Invitrogen), B-27 (Invitrogen) and 1% FBS in each maintain medium with medium change every 2 days. We induced neural retina differentiation of somatic stem cells in two manners: using a cocktail of exogenous factors or a miRNA inhibitor. By the first manner, cells were cultured in the differentiation medium supplemented with 100 ng/ml Dkk-1 (R&D systems), 10 ng/ml Noggin (R&D systems), 10 ng/ml IGF-1 (Peprotech) and 5 ng/ml bFGF (Peprotech). By the other one, we cultured cells in the differentiation medium and repeatedly treated anti-miR-203 (Ambion) to the cells every 7 days. After 3 weeks, cells were maintained without the cocktail of exogenous factors or anti-miR-203.

### Computational analysis

Predictions of hsa-miR-203 (GUGAAAUGUUUAG GACCACUAG) targets were performed using three different online miRNA target prediction programs, namely, *TargetScanHuman* (Release 5.2), *miRanda* (Release August 2010) and *DIANA microT* (Version 3.0). For hsa-miR-203-3p, TargetScan predicted 3916 target genes, miRanda predicted 9208 targets, and DIANA predicted 3836 targets.

### Cell transfection

Before the transfection of miRNA inhibitor using *DharmaFECT* Transfection Reagent (Thermo Scientific), 3 × 10^5^ cells were seeded in 60-mm dishes. Next day, a mixture of transfection reagent and anti-miRs was prepared and added to the cells according to the manufacturer's protocol. Cells were transfected with anti-miR-203 (Anti-miR miRNA Inhibitor; Ambion) at a final concentration of 30 nM. AllStars Negative Control siRNA (QIAGEN), which is thoroughly tested and validated non-silencing siRNA with no homology to any known mammalian gene, was used as a negative control siRNA. One day after transfection, medium was replaced with the differentiation medium. Repeat transfections were performed every 7 days up to three times.

### Cloning and luciferase assay

A fragment of the 3′-UTR containing the predicted miR-203 target binding sequence (CATTTCA) was amplified by RT-PCR. These 3′-UTR fragments were cloned into *pmirGLO Dual-Luciferase miRNA Target Expression Vector* (Promega) using *T4 DNA Ligase* (Invitrogen). For the luciferase assay, 1 μg of reporter construct was co-transfected with miR-203 or a negative control at a final concentration of 30 nM into 293T cells using *Lipofectamine 2000* (Invitrogen) according to the manufacturer's protocol. After 48 hours, Firefly and Renilla luciferase activities were measured using *Dual-Luciferase Reporter Assay System* (Promega) according to the manufacturer's instructions.

### Statistical analysis

All of the experiments were conducted at least in triplicate, and the results are expressed as the mean ± SD. Statistical analyses were conducted via Student's *t*-test. A value of *P* < 0.05 was considered significant (*P < 0.05; **P < 0.01; ***P < 0.001).

## SUPPLEMENTARY FIGURES AND TABLES


