# Supplementary material for: Direct cell fate conversion of human somatic stem cells into cone and rod photoreceptor-like cells by inhibition of microRNA-203
Source: Oncotarget. 2016 Jun 7;7(27):42139–49. doi: 10.18632/oncotarget.9882 (PMC5173122; doi:10.18632/oncotarget.9882)
Supplement: Supplementary file 1 [file oncotarget-07-42139-s001.pdf]

Direct cell fate conversion of human somatic stem cells into cone and rod photoreceptor-like cells by inhibition of microRNA-203

Supplementary Materials

MATERIALS AND METHODS

Primary cell culture

Briefly, human amniotic tissue was washed with PBS and incubated with 0.05% trypsin-EDTA (Invitrogen) for one hour. The single cells were collected and cultured in K-SFM supplemented with 0.031 µg/µl human recombinant EGF (Invitrogen), 12.4 mg/ml bovine pituitary extract and 10% FBS. For human UCB-MSCs, the mononuclear cells were separated from the umbilical cord blood using Ficoll-Paque TM PLUS (Amersham Bioscience) and were suspended in a Dulbecco's modified Eagle's medium (DMEM; Gibco), containing 20% FBS (Hyclone), 100 I/ml penicillin (Gibco), 100 mg/ml streptomycin (Gibco), 2 mM L-glutamine (Invitrogen), and 1 mM sodium pyruvate (Invitrogen).

Retina tissue and RT-PCR

For RT-PCR analysis, total RNA was extracted from AESCs and UCB-MSCs using TRIzol reagent according to the manufacturer's instructions. The total RNA source of normal human retina purchased from Clontech Laboratories, Inc. (Cat.# 636579; Lot.# 1005010; pooled from 6 male and female Caucasians, aged between 44 and 79). The cDNA synthesis and RT-PCR experiments were performed as described previously [38]. Each gene expression was normalized by GAPDH as housekeeping control and relative gene expression levels were calculated using $2^{-\Delta\Delta Ct}$ method. All quantitative RT-PCR experiments were performed using three technical and three independent biological replicates. Primer sequences are listed in Table S5.

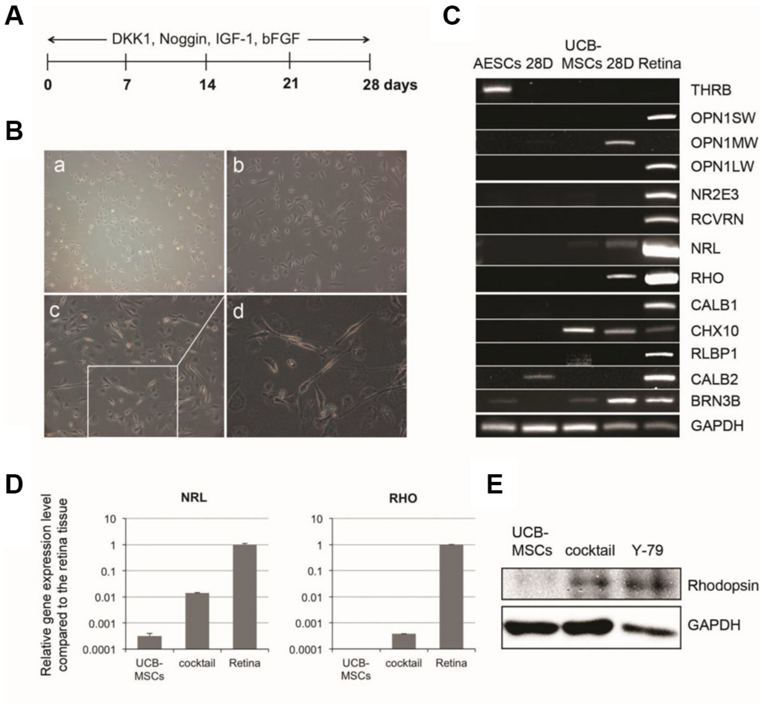

Supplementary Figure S1: Somatic stem cells can give rise to neural retina subtypes by the retina differentiation medium. (**A**) A differentiation scheme using DKK1, Noggin, IGF-1 and bFGF results in the neural retina differentiation of UCB-MSCs and AESCs in 28 days. (**B**) Phase-contrast images of AESCs during the differentiation are shown at different time points, namely 0 days (a), 14 days (b) and 28 days (c and d; d is a magnification of c). (**C**) An RT-PCR analysis shows the neural retina-specific gene expression pattern of AESCs and UCB-MSCs before and after 28 days of differentiation (28D) compared to human retina tissue (Retina). (**D**) A quantitative RT-PCR analysis shows the relative gene expression levels of NRL and RHO in UCB-MSCs, the cocktail-induced PR-like cells and human retina tissue. (**E**) Western blot analysis shows the expression of Rhodopsin in the cocktail-induced PR-like cells and the retinoblastoma cell line Y-79.

Supplementary Table S1: Target human genes of 20 microRNAs by three different programs

Systematic Name	Target genes by miRanda	Target genes by TargetScan	Target genes by DIANA	Expression level compared to miR-203 in AESCs
**hsa-miR-1**	CRX, OTX2, NEUROD1	CRX, OTX2, NEUROD1, THRB	CRX, OTX2, THRB, NEUROD1, RORB	0.8
**hsa-miR-143**	CRX, THRB	CRX, THRB	CRX, THRB	0.6
**hsa-miR-150**	CRX, RORB, NRL, NR2E3	CRX, RORB, NRL, THRB, NR2E3	CRX, RORB, NRL, THRB	0.7
**hsa-miR-185**	CRX, NR2E3	CRX, NEUROD1	CRX, THRB	0.7
**hsa-miR-192**	CRX	CRX	CRX	0.6
**hsa-miR-203**	**CRX, NEUROD1, RORB, NRL, THRB**	**CRX, NEUROD1, RORB, NRL, THRB**	**CRX, NEUROD1, RORB, THRB**	**1**
**hsa-miR-206**	CRX, OTX2, NEUROD1	CRX, OTX2, NEUROD1, THRB	CRX, OTX2, THRB, NEUROD1, RORB	0.7
**hsa-miR-215**	CRX	CRX	CRX	0.8
**hsa-miR-221**	CRX, RORB, THRB	CRX, RORB, THRB	CRX, NEUROD1, RORB, THRB	2.5
**hsa-miR-222**	CRX, RORB, THRB	CRX, RORB, THRB	CRX, NEUROD1, RORB, THRB	0.9
**hsa-miR-296-3p**	CRX, NRL	CRX, NRL	CRX, THRB	0.5
**hsa-miR-302e**	CRX, NEUROD1	CRX, NEUROD1, RORB	NEUROD1, RORB	0.5
**hsa-miR-339-5p**	CRX, THRB, NR2E3	CRX, THRB, NR2E3	CRX, NEUROD1	0.7
**hsa-miR-491-5p**	CRX	CRX, THRB	CRX	0.6
**hsa-miR-520a-3p**	CRX, NEUROD1	CRX, NEUROD1, RORB	NEUROD1, RORB	0.5
**hsa-miR-520b**	CRX, NEUROD1	CRX, NEUROD1, RORB	NEUROD1, RORB	0.8
**hsa-miR-520c-3p**	CRX, NEUROD1	CRX, NEUROD1, RORB	NEUROD1, RORB	0.5
**hsa-miR-520e**	CRX, NEUROD1	CRX, NEUROD1, RORB	NEUROD1, RORB	0.8
**hsa-miR-613**	CRX, OTX2, NEUROD1	CRX, OTX2, NEUROD1, THRB	CRX, OTX2, NEUROD1, THRB	0.5
**hsa-miR-653**	CRX, NEUROD1, NR2E3	CRX, NEUROD1, NR2E3	CRX, NEUROD1, RORB	0.6

Supplementary Table S2: hsa-miR-203 targets scored by TargetScan

Target gene	Target site	Total context score	Aggregate P^{CT}
DKK1	1	−0.26	0.12
CRX	2	−0.23	0.17
RORβ	1	−0.13	0.13
NEUROD1	1	−0.22	0.14
NRL	1	−0.15	< 0.1
THRB	2	−0.22	0.46

Of 3916 predicted target genes of has-miR-203.

Supplementary Table S3: hsa-miR-203 targets scored by miRanda

Target gene	Target sites	mirSVR score	PhastCons score
DKK1	346–368	−0.9573	0.6298
	549–570	−0.1016	0.6379
CRX	1161–1182	−0.0051	0.5662
	1468–1489	−0.0008	0.5342
	1564–1583	−0.6578	0.5676
	3158–3179	−0.0024	0.5575
RORβ	944–965	−0.0287	0.5990
	1058–1079	−0.4580	0.5546
NEUROD1	511–532	−0.1716	0.7584
	683–704	−0.9647	0.5915
NRL	446	−0.1702	0.5694
THRB	32	−0.0634	0.5984
	1715	−0.0013	0.6117
	2175	−0.0008	0.5386
	4771	−0.0056	0.5281
	5618	−0.0241	0.5569

Of 9208 predicted target genes of has-miR-203.

Supplementary Table S4: hsa-miR-203 targets scored by DIANA

Target gene	Target sites	miTG score	SNR	Precision
DKK1	359−367	2.16	1.05	0.04
CRX	1173−1181<br>1480−1488<br>1574−1582	7.84	1.05	0.2
RORβ	1070−1078	2.22	1.05	0.11
NEUROD1	695−703	4.58	1.17	0.18
THRB	24−52	2.25	1.14	0.11

Of 3836 predicted target genes of has-miR-203.

Supplementary Table S5: Primer sequences

Gene name	Product (cDNA)	Product (gDNA)	Sequence (forward)	Sequence (reverse)
DKK1	157	275	tccgaggagaaattgaggaa	cctgaggcacagtctgatga
DKK3	238	238	ttcatccagcagtgttgctc	ggtgtggggtagtggagaga
CHX10 (VSX2)	169	X	gagaaggcattcaacgaagc	catactccgccatgacactg
PAX6	255	1140	cagctcggtggtgtctttg	agtcgctactctcggttta
RX (RAX, RX1)	200	200	gtccctaagcgtgctttcag	catgccagggtcttggtact
OTX2	429	X	caacagcagaatggaggtca	ctgggtggaaagagagaagctg
CRX	187	3112	gtgaggaggtggctctgaag	acatctgtggagggtcttgg
RORB	196	X	tttaggaggagccagcagaa	tctgcacctcagcatacagg
NEUROD1	152	1767	tagacctgctagcccctcag	ctcgctgtacgatttggtca
THRB (TRB2)	225	X	agctgaaaaatgggggtctt	tcacgtggtgttttcggtaa
OPN1MW	192	2179	gctacaccgtctccctgtgt	acctgctccaaccaaagatg
NR2E3	151	3585	aggaccagtcccaagtgatg	cctatggtcttgcggaaaaa
Recoverin	150	2934	agctccttccagacgatgaa	caaactggatcagtcgcaga
NRL	222	222	ccaagaagtccccaagacaa	aagtctcacctcagccctca
Rhodopsin	186	1967	tcatggtcctaggtggcttc	ggaagttgctcatgggctta
Calbindin (CALB, CALB1)	153	X	tttcgagatctggctccatt	tgcccatactgatccacaaa
PRKCA (PKC-alpha)	172	X	cctaaaggctgaggttgctg	atttagtgtggagcggatgg
Calretinin (CALB2)	208	2190	gctccaggaatacacccaaa	cagctcatgctcgtcaatgt
RLBP1 (CRALBP)	226	1901	agcccgatttaacggaaact	ttggacctgggttcaagttc
STX1A (HPC-1)	221	X	gaccgcttcatggatgagtt	atggactgctcgatgctctt
BRN3B (POU4F2)	219	219	agccggtgagaatgtgaaac	tgaacacgggtgatgtctgt
